# Coxe on the Treatment of Membranous, or True Croup

**Published:** 1859-01

**Authors:** 


*Coxe on the Treatment of Membranous, or True Croup.*—When innocent persons are incarcerated on the suspicion of a crime, their claims on our sympathy are undoubted, and when a helpless child, innocent of the slightest predilection towards membranous croup, is subjected to the dangerous, not to say disagreeable, processes which are recommended by Dr. Coxe, we cannot close our heart to its cause, and must express our sense of the injustice of such treatment. Dr. Edward Jenner Coxe, Visiting Physican, Charity Hospital, New Orleans, has communicated a case of croup, treated successfully, according to his plan, to the *Boston Journal*, which must astonish some Bostonians, who have gloried in their townsman's, Dr. Ware's, labors in this disease. We consider the mode of treatment employed by Dr. Coxe too pernicious to pass without comment, especially as the Doctor ascribes a success to his plan, which is entirely due to a blunder of diagnosis. We apprehend, indeed, that the blunder has been made not once, but one hundred times, for the article says: "Now, when I can conscientiously assert that out of one hundred cases of true croup, which I have treated in Philadelphia and this city, but one has died, it is not to be considered strange that, without a desire of boasting, my faith in the mode of using the remedies, powerful as I know them to be, should be strong." Now, Dr. Coxe has treated a hundred cases of *true* croup with but one death: by true croup he can mean nothing but membranous croup; if his diagnosis be correct, M. Trousseau may cease his advocacy of tracheotomy, Dr. Ware will be forgotten, M. Bouchet's ingenious invention for tubing the glottis, is useless; for look at the statistics in regard to this disease in the hands of the most eminent practitioners of Europe and America. In the admirable article on Pseudo-membranous Laryngitis in Meigs' Diseases of Children, we have it stated, that Dr. Bond, of New York, says that of sixteen cases, seven died; of twenty-two cases seen by Dr. Meigs, ten died; of sixty cases observed by M. Ferraud, in the villages about La Chapille-Véronge, not a single one escaped; M. Guersent, after a careful consideration of the statements of different authors, says: in fact, the croup is one of the most dangerous of all diseases, and is generally fatal. He adds, that he has seen at least one hundred cases of spasmodic croup, (the same number cured by Dr. Coxe,) without a single death, while of ten children attacked with true croup, it is scarcely possible to save two. We conclude that the cases of spasmodic croup seen by M. Guersant, were hardly subjected to the active medication employed by Dr. Coxe. We will now give the case which forms the basis of Dr. Coxe's article:

H. L., a boy aged 13 years, of usual size, and healthy, for years has not had an attack of croup, although very subject to it some years since, when living in Mobile. Nov. 18th, he complained of feeling chilly, and an occasional cough, not hoarse or croupy. His mother, supposing it to be a slight cold, put him to bed early. Toward midnight, he awoke his brother in the same room, by his constant cough, which was dry and hoarse, with a noisy inspiration. After some time, the mother was called, who, recognizing the disease, took him to her room, gave him several doses of sweet oil, nothing else being at hand, and, finding matters getting worse, she sent for me. At 3 o'clock, A. M., I was there, and found him laboring for breath, with the characteristic inspiration and cough, which once heard, never can be mistaken. He constantly complained of his throat, he could scarcely breathe, and swallowed with difficulty what was given. Apprised, when called, of the nature of the sickness, I took with me a bottle of hive syrup, and a small box of medicines which I keep for such occasions, and always find beneficial. The skin was hot and dry, the pulse tense, frequent and moderately full, the face flushed, the eyes injected, the boy restless, and evincing every sign of great distress. To act vigorously and promptly was imperative. At once I poured down his throat a dessertspoonful of hive syrup; and, as soon as ready, ten grains of calomel, six or seven of tartar emetic, and half a teaspoonful of ipecacuanha, were mixed with half a tablespoonful of hive syrup, and poured down his throat by myself. Several times, before vomiting occurred freely, although he had two or three times brought up with a hard cough, pieces of tough phlegm, the same mixture of calomel, tartar emetic, and ipecacuanha, in similar quantities, was given; in one or two of the last, having prepared a strong solution of nitrate of potash, about four ounces, in which was dissolved about six grains of tartar emetic, some of this was added. This last was repeated several times, in addition to the mixture. Although vomiting with hard straining occurred several times, accompanied by cough and tough phlegm, I was not satisfied.

The symptoms continued severe, and I began to fear I should not succeed, when I took about fourteen ounces of blood from the arm, which sensibly affected the pulse, caused a feeling of faintness, at least he fell on his side, had a more free vomiting, and, best of all, an evidently fuller and freer inspiration, with less of the stridulous sound. More of the same mixture was given at longer intervals; a mustard poultice was applied to the throat, and he was allowed a little rest. As I watched him closely, while he was lying quietly, I found his breathing more natural, and his croup, which occurred from time to time, softer, with but little of the peculiar sound. I waited quietly some time, and was satisfied he was asleep, and safe; I then mixed another dose of calomel, tart. emetic, ipecac., and solution of nitrate potassa and tart. antimony, and gave directions to the mother to give it, in case he had any return of cough, or difficulty of breathing; but if he continued to sleep, not to awake him, but as soon as he did awake, to give it to him. A little before six o'clock, I was in my bed at home. At half-past eight, A. M., I saw him again, when I found he was doing well; that he had slept more than an hour, awoke, took the dose that had been left, and dropped asleep again. He was awake when I called; he had coughed several times during my absence. I made him cough several times, and breathe freely, to satisfy myself. There was still some of the dryness of cough, and peculiarity of inspiration, with soreness of the throat. I allowed him a little sweetened milk and water, and a lemonade of gum arabic in flaxseed tea, for drink. The following was ordered:—℞. Nit potassa, a drachm and a half; tart. antimony et potassa, two grs.; tr. verat. virid., fifteen drops; syr. morphiæ, six drachms; aquæ, two ounces. Dose, one teaspoonful every hour, until again seen. I omitted mentioning that his bowels were twice opened during the night, and once this morning. For one day, he was kept in bed; the next day he was on the sofa, in the parlor; the mixture, and occasional small doses of hive syrup, being given during the day, as the cough continued, and the inspiration was not free from the peculiar sound. In a few days he was about the house, coughing occasionally, and taking hive syrup and paregoric at bed-time. Had there been any paregoric in the house on the first night, when he fell asleep, I should have given him one or more full doses with the other medicines, for the express object of making him sleep soundly for many hours, which is generally my rule, and always works well.

In conclusion, I would remark, that for the ordinary diseases of infants or children, I am not partial to much medication, if possible to be avoided; but in treating croup, as I do not wish my patients to die, I know no limit, either as to the quantity or frequency of the doses of those articles named, in which I put my trust. When I assert, as a solemn truth, that I have yet to see the first case in which the treatment laid down has produced any injurious subsequent effects, why should others so strenuously oppose this course of treatment, the only one which, in my opinion, is adequate to effect a cure, and prevent the dire necessity of resorting to tracheotomy. The fact, that my patient took even more of the active remedies than has been noted, and that no immediate or subsequent ill effects, but a perfect cure, resulted, is worthy of consideration.

We have copied the full report of this case; but at the first glance, our readers will not be able to appreciate the quantity of medicine taken by this devoted boy, between 3 A. M. and 6 A. M.

One tablespoonful of hive syrup, ten grains of calomel, seven grains of tartar emetic, one-half a teaspoonful of ipecac., and this multiplied by *several times*, of which several times, the last one or two, there was added some of a strong solution of nitrate of potash, in which was dissolved about six grains of tartar emetic. Here we have another several times, for "this last was repeated *several times*, in addition to the mixture." After this, "more of the same mixture was given at longer intervals; a mustard poultice was applied to the throat, *and he was allowed a little rest*" *!!!*

This is the treatment for the first three hours; we are unable to calculate the exact amount of drugs which were taken, on account of the indefiniteness of the author's statement; but at the lowest estimate, he must have taken 6 *tablespoonsful of hive syrup*, 3 *teaspoonsful of ipecacuhana*, 60 *grains of calomel*, 60 *grains, at least, of tartar emetic, and, perhaps, two ounces of nitrate of potash.* In addition to this, fourteen ounces of blood were taken from the arm.

For what, we now enquire, was all this medication employed? A glance at the symptoms is sufficient to show that it was merely a case of catarrhal, false, or spasmodic laryngitis, which would have progressed favorably with the most simple remedies, if any were employed. It is almost unnecessary to discuss this point; there is absolutely nothing in the history of the case which would lead to the suspicion of the danger of false membrane. The attack was instantaneous; there was no initiatory fever; the fauces were not examined; there were no membranous formations discovered in the expectoration, nothing, in fact, which would lead us to suspect true croup, and everything which would indicate the true nature of the difficulty. In regard to the hundred cases treated by Dr. Coxe, with but one fatal issue, we are confident, if this be an example, that we can produce old women, who have seen, we were about to say, thousands of such cases, and the only difference would be, that it would be difficult to find one who had seen one result fatally.

We cannot too emphatically condemn such fearful medication, *in any disease*, as pernicious in the extreme, and especially so in a case where it cannot be that any vigorous measures were called for. In such cases, even when the bronchial passages are almost occluded with mucus, we seldom find it necessary to do more than dislodge it occasionally with a mild emetic of syrup of ipecacuhanna, or, what is still better, one of simple powdered alum, as recommended by Dr. Meigs. We hope that Dr. Coxe will learn, that in cases, such as he has described, there will be no "dire necessity of resorting to tracheotomy," even should he become a passive spectator of the course of the disease; and that such a course would be infinitely preferable to the one which he advocates so insanely.

---

*The New York Medical Press: A Weekly Journal of Medicine, Surgery, and the Collateral Sciences.* Edited by J. L. Kiernan, A. B., M. D., and W. O'Meager, M. D.

We have received the first number of this new weekly, published in New York, and are pleased with its appearance, and the matter which it contains. Within the last year, we have had three new weekly journals, and this makes the second in the city of New York. We put it with pleasure on our exchange list.

---

*Communications Received.*—We have received communications from Drs. Merriam, Spencer, and Crooks, which will receive an early insertion; also, a number of books and pamphlets, which we will notice at the earliest opportunity.

